# Identification of a Cytokine Biomarker for Prognostic Modeling of Breast Cancer–Related Lymphedema

**DOI:** 10.1158/2767-9764.CRC-25-0541

**Published:** 2026-03-03

**Authors:** Alison J. Wu, Neil Lin, Jie Su, Cherie Lin, Madison-Shira Hossack, Wei Shi, Farnoosh Abbas-Aghababazadeh, Wei Xu, Benjamin Haibe-Kains, Simona F. Shaitelman, Melissa B. Aldrich, Fei-Fei Liu, Jennifer Y.Y. Kwan

**Affiliations:** 1 MD Program, Temerty Faculty of Medicine, University of Toronto, Toronto, Canada.; 2 Research Institute, Princess Margaret Cancer Centre, University Health Network, Toronto, Canada.; 3 Institute of Medical Science, Temerty Faculty of Medicine, University of Toronto, Toronto, Canada.; 4 Biostatistics Division, Princess Margaret Cancer Centre, Toronto, Canada.; 5 Princess Margaret Bioinformatics and Computational Genomics Laboratory, University Health Network, Toronto, Canada.; 6 Department of Computer Science, University of Toronto, Toronto, Canada.; 7 Ontario Institute for Cancer Research, Toronto, Canada.; 8 Vector Institute for Artificial Intelligence, Toronto, Canada.; 9 Department of Radiation Oncology, MD Anderson Cancer Center, Houston, Texas.; 10 The University of Texas MD Anderson Cancer Center UTHealth Houston Graduate School of Biomedical Sciences, Houston, Texas.; 11 UTHealth Brown Foundation Institute of Molecular Medicine for the Prevention of Human Diseases, Houston, Texas.; 12 Department of Radiation Oncology, Temerty Faculty of Medicine, University of Toronto, Toronto, Canada.; 13 Department of Medical Biophysics, Temerty Faculty of Medicine, University of Toronto, Toronto, Canada.; 14 Radiation Medicine Program, Princess Margaret Cancer Centre, Toronto, Canada.

## Abstract

Lymphedema is a chronic complication of breast cancer treatment, and early intervention is crucial to reduce morbidity. This study evaluated the role of blood-based cytokine biomarkers in the prognostication of breast cancer–related lymphedema (BCRL) to improve risk prediction. A secondary analysis of inflammatory biomarkers for BCRL was performed using a previously published cohort of 147 patients with breast cancer who had undergone serum cytokine profiling during their treatment at the Princess Margaret Cancer Centre from 2010 to 2014. Prognostic cytokine variables for lymphedema were selected by regression analysis and independence from known clinical risk factors. Regression-based modeling was employed to integrate prognostic variables for the prediction of lymphedema occurrence. We identified the immunostimulatory cytokine IFNα2A as a potential biomarker for lymphedema development [OR, 3.10; 95% confidence interval (CI), 1.05–9.51; *P* = 0.042], independent from known clinical risk factors. Furthermore, Kaplan–Meier analysis demonstrated 3-year lymphedema-free survival of 95% (90%–100%) versus 85% (77%–94%) for below versus above median concentrations of IFNα2A (*P* = 0.026). In combination with an established clinical risk regression-based model, patients identified as high risk based on clinical factors alone were able to be correctly reclassified as low risk by IFNα2A in 31% (eight of 26) of cases. Our combined logistic regression model using both IFNα2A and clinical risk score achieved an AUC of 0.895 (95% CI, 0.796–0.971) and Brier score of 0.101 (95% CI, 0.061–0.149), representing a favorable improvement compared with the logistic regression model using clinical risk factors alone. IFNα2A in combination with established clinical risk factors may be useful for improving BCRL prognostication.

**Significance:**

The cytokine IFNα2A was identified as a potentially complementary biomarker to improve the stratification of high- and low-risk patients for BCRL. This will help enable earlier intervention to reduce long-term morbidity for those at high risk for lymphedema while minimizing burdensome interventions for those at low risk.

## Introduction

Lymphedema is a debilitating, incurable complication of breast cancer treatment, characterized by progressive swelling of the arm, shoulder, neck, trunk, or breast. It typically occurs after radiotherapy or axillary surgical interventions (1), during which nearby lymph nodes are damaged or removed. This leads to impairment of lymphatic drainage (2) and an accumulation of extracellular fluid in surrounding tissues (3), resulting in an enlargement in limb circumference, chronic inflammation (4), tissue fibrosis, and abnormal adipocyte proliferation (5).

This lymphatic dysfunction and related pathologic changes pose a substantial burden on many patients; indeed, the median incidence of breast cancer–related lymphedema (BCRL) within 3 years of treatment is estimated to be 20%, with 10% to 64% of such individuals reporting upper body symptoms (6). In particular, patients of older age, those who identify as Black or Hispanic, and those who received neoadjuvant chemotherapy have been shown to be at greater risk of BCRL (7). Patients with BCRL often suffer from a spectrum of physical, medical, and psychosocial consequences that greatly affect their quality of life and day-to-day functioning. Potential issues include recurrent soft-tissue infections, discomfort, disability, and psychologic distress related to body image and social relationships. Although there are conservative physical strategies and emerging surgical procedures that can manage BCRL, there is currently no cure. Maintenance treatment for BCRL—which is often burdensome and time-consuming—must be continued on a lifelong basis (8).

With the onset and severity of BCRL varying widely among patients (9), there is an increasing interest in identifying biomarkers to predict the risk of developing this condition. Inflammatory cytokines in particular show considerable promise as prognostic biomarkers given that they are involved in numerous molecular mechanisms associated with the pathophysiology of BCRL. Current evidence shows that the early phase of acute inflammation caused by radiotherapy or surgery results in the activation and infiltration of immune cells, including CD8^+^ T cells, T-helper cells, dendritic cells, macrophages, and neutrophils. This occurs alongside a local accumulation of interstitial fluid; eventually, the inability of the lymphatic system to accommodate this fluid overload incites a cascade of chronic inflammatory events (10). Such events include the impaired transit and excessive accumulation of activated inflammatory/immune cells, as well as the release of cytokines within the edematous environment (11), leading to chronic changes that irreversibly transform surrounding tissues through fibrosis and excess adipose deposition (12). These late effects caused by an inflammatory cascade mean that lymphedema can be diagnosed months or even years after treatment.

Several recent studies have investigated the presence and potential roles of specific cytokines in lymphedematous tissues from murine models or human samples. In a study using a specific bioassay to conduct transcriptional profiling of human skin, numerous cytokine genes, including *IL4*, *IL6*, *IL10*, and *IL13*, were found to be upregulated in lymphedema samples (13). In another study of patients who underwent breast cancer surgery, common variants in the cytokine genes *IL4*, *IL10*, and *NFKB2* were significantly associated with BCRL (14). Furthermore, in a mouse tail model of lymphedema, the inhibition of Th2 cytokines IL4 and IL13 prevented the T-cell inflammatory response, which led to decreased fibrosis and improved lymphatic function (15). In addition to IL4 and IL13, the cytokine TGFβ1 has also been shown to be a key promoter of fibrotic remodeling and collagen deposition in secondary lymphedema (16–18). Other cytokines such as G-CSF, GM-CSF, IFNα, IL10, IL12p40, IL15, IL17A, IL1β, IL2, IL3, IL6, and MIP1β have been found to be elevated prior to cancer treatment in patients who later developed BCRL at 12 months after radiotherapy compared with those who did not develop BCRL (19, 20). Together, these findings strongly suggest that differences in cytokine expression play an important role in the development of secondary lymphedema.

Previously, we developed a multivariable linear regression model that used age, body mass index (BMI), mammographic breast density, number of pathologic lymph nodes, and axillary lymph node dissection (ALND) to predict the development of BCRL (21). In this current study, we further investigated the association of 17 baseline serum cytokines with the occurrence of BCRL in a previously reported cohort of patients with mainly early-stage breast cancer prospectively evaluated for cytokine levels and the development of fatigue during radiotherapy (22). We sought to identify the most important cytokine biomarkers among these 17 that can predict lymphedema occurrence independently of clinical factors, and in doing so, we aimed to develop more accurate models for personalized prognostication of BCRL. To our knowledge, this is the first study to integrate a cytokine biomarker with a lymphedema risk prediction model. These models have the potential to be used by healthcare professionals to more accurately predict BCRL risk for patients prior to radiotherapy and enable clinicians to implement early interventions in recognition of lymphedema risk. Thus, our work will help enable better prognostication of BCRL for early treatment and prevention when necessary and contribute to a growing body of knowledge surrounding biological markers and immune-targeted therapies for secondary lymphedema.

## Materials and Methods

### Research ethics

This secondary analysis was approved by the Research Ethics Board of the University Health Network. There was no patient or public involvement in the design, conduct, reporting, interpretation, or dissemination of this study. The IFNα2 dataset from the University of Texas MD Anderson Cancer Center was registered with ClinicalTrials.gov (NCT number: 02949726).

### Study population

We performed a secondary analysis of a previously reported prospective cohort study on inflammatory biomarkers in patients with breast cancer undergoing adjuvant radiotherapy in a tertiary care setting (22); a subset of this cohort has also been analyzed for noncytokine biomarkers of lymphedema (23). A total of 152 patients with breast cancer were recruited to this longitudinal cohort study between February 2010 and July 2014 at the Princess Margaret Cancer Centre, University Health Network in Toronto, Ontario, Canada (22). All study participants underwent primary tumor resection and radiotherapy (with or without chemotherapy), were aged 18 years or older, and had either ductal carcinoma *in situ*, invasive ductal carcinoma, or lobular carcinoma confirmed by histology (22). Patients with hematologic conditions or previous bone marrow transplant were excluded (22) to avoid confounding from underlying immune dysregulation and chronic inflammation which may affect the results of cytokine profiling. The sample size was predetermined based on the prior prospectively recruited cohort. Findings were subsequently compared with an independent dataset.

Demographic data and clinical characteristics for all participants were collected at enrollment from electronic health records, illustrating a population representative of patients with mainly early-stage breast cancer within a Canadian academic hospital setting. Data included age, BMI, tumor characteristics (such as tumor and nodal staging), and cancer treatment characteristics. For this study, we performed a secondary review of lymphedema-associated risk factors, including mammographic breast density assessed with the Breast Imaging-Reporting and Data System (BI-RADS), and lymphedema outcomes. The range of patient follow-up time was between 0.2 and 11.8 years from the date of cancer surgery. Data collection was completed and independently reviewed by W. Shi, M.-S. Hossack, and J.Y.Y. Kwan.

### Lymphedema measurement

Lymphedema was clinically diagnosed by a medical provider and quantified as an increase in arm volume from baseline in the affected arm compared with the contralateral unaffected arm. Circumferential arm measurements were conducted with a standardized protocol, with measurements conducted at seven standard points. Volume was calculated based on these measurements and recorded as both absolute (mL) and percent (%) increase. Measurements were routinely recorded by trained physiotherapists and occupational therapists using standard dictation templates and collected for this analysis.

### Serum cytokine quantification

Serum samples were collected via phlebotomy from 147 of the 152 patients. Phlebotomies were conducted within the same 4-hour period for each patient to reduce the effect of diurnal variations (24). Baseline (preradiotherapy) cytokine levels were selected for analysis in this study.

As previously reported, the levels of 17 cytokines were measured to evaluate their association with fatigue (22), including C-reactive protein (CRP), IFNγ, IFNα2A, IL10, IL17A, IL1b, IL1 receptor antagonist (IL1RA), IL4, IL6, interferon gamma–induced protein 10 (IP10), monocyte chemoattractant protein-1 (MCP-1), matrix metalloproteinases-2 (MMP2), matrix metalloproteinases-9 (MMP9), stromal cell-derived factor-1α (SDF1α), TGFβ1, TNF receptor II (TNFRII), and TNFα (Supplementary Table S1). Cytokine levels were measured using custom multiplexed electrochemiluminescence immunoassays (Meso Scale Discovery) and recorded as concentrations (pg/mL). The protocol was described by Shi and colleagues (22). As previously described in the literature, many of these cytokines have also been shown to affect lymphatic function; therefore, we selected them as possible predictors for lymphedema risk modeling in this study.

### Biostatistical analysis and model development

Five participants were excluded from our dataset of 152 patients given that they lacked baseline serum cytokine measurements, yielding a final sample size of 147 patients. Among these 147 patients, 24 were lacking BMI data and 38 were lacking mammographic breast density data. These missing data were substituted by mean imputation (i.e., mean BMI of 26.5 kg/m^2^ and mean BI-RADS score of 2.5 were imputed for missing values).

Data analyses were performed using SAS, version 9.4 by SAS Institute Inc. (RRID: SCR_008567) and R, version 4.4.1 by The R Foundation for Statistical Computing. Descriptive statistics were performed on this dataset. Frequency (percentage) is provided for categorical variables whereas mean (SD), median (Q1, Q3), and range (min, max) are presented for continuous variables.

Nonparametric Wilcoxon rank-sum testing was applied to determine the associations between candidate cytokines and established clinical risk factors, defined as per our prior study (21). Cytokines without significant association with the clinical risk assessment (*P* > 0.05) were selected for univariate logistic regression. A Kaplan–Meier curve of 3-year lymphedema-free survival (LFS) with a log-rank test *P* value was generated using a median cytokine concentration threshold. Net reclassification improvement was used to assess the performance of our integrated two-step clinical risk model.

Multivariable modeling was performed using logistic regression, yielding binary outcomes for lymphedema presence (i.e., lymphedema present or absent). Variables assessed included the five-factor clinical risk model score (21) and IFNα2A. The five factors in the clinical risk score were age, BMI, mammographic breast density, number of pathologic lymph nodes, and ALND, as shown in the equation below from our previous study (21):

Lymphedema volume = -329 + (4 × age) + (10 × BMI) - (37 × mammographic breast density) + (13 × no. of pathologic lymph nodes) + (99 × ALND treatment use)

We calculated the five-factor clinical risk scores for each study participant, which were dichotomized as a high risk (score >200) or low risk (score ≤200) based on the prior study (21, 23). IFNα2A was dichotomized by median threshold. We then utilized one iteration of fivefold cross-validation to train and evaluate performance for prediction of lymphedema occurrence. The multivariable logistic regression model was developed from the scikit-learn library (RRID: SCR_002577) in Python. Training and performance evaluation using fivefold cross-validation was completed. Performance metrics [e.g., area under the receiver operating characteristic curve (AUROC), sensitivity, specificity, and Brier score] were calculated and used to plot calibration and ROC curves with the Matplotlib library (RRID: SCR_008624). Nonparametric 95% confidence intervals (CI) for all metrics were determined with bootstrapping, involving 1,000 iterations of resampling with replacement.

### IFNα2 comparison dataset

We performed a secondary analysis of an independent cohort of patients with breast cancer from the University of Texas MD Anderson Cancer Center. This cohort has been previously described including its eligibility criteria (20). A total of 62 study subjects with preradiotherapy measurements of plasma IFNα2 were eligible for this analysis. The plasma IFNα2 levels at this timepoint have not been previously published. Plasma IFNα2 was quantified using the Human Cytokine/Chemokine/Growth Factor Panel A Magnetic Bead Panel 96-well plate assay (cat. #HCYTA-60K, MilliporeSigma) as per the manufacturer’s protocol. Subjects were categorized by their lymphedema status at 18 months after cancer surgery. A two-sided Mann–Whitney test was used to evaluate the difference between subjects who did or did not develop lymphedema; *P* < 0.05 was selected as the significance level.

## Results

### Patient characteristics

The clinical characteristics of these 147 patients have already been published (22), including BMI, mammographic breast density, and presence of lymphedema for a subgroup of the study population (Table 1; ref. 23). The mean age of the study population was 56.1 years, with the mean BMI being 26.5 kg/m^2^. The distribution of mammographic breast densities included 51% (56) with lower-density and 49% (54) with higher-density breasts. The majority (70.1%) of patients (104 of 147) were diagnosed with Tis/T1 breast cancer. The mean number of pathologic nodes was 0.9, with 5.4 nodes removed on average. The majority (89.1%) of patients (131 of 147) had lumpectomy as their primary surgery, and 23.1% of patients (34 of 147) underwent ALND. Hypofractionated radiation (42.4 Gy/16 fractions) was delivered to 68.7% of patients (101 of 147), whereas all other patients received conventional fractionated radiation (50 Gy/25 fractions). Nearly half (48.2%) of the patients (71 of 147) received a radiotherapy boost. More than a third (35.4%) of patients (52 of 147) received neoadjuvant or adjuvant chemotherapy prior to radiotherapy, and 63.9% of patients (94 of 147) received adjuvant hormone therapy. Lymphedema was observed in 10.2% of patients (15 of 147). The median follow-up time was 4.3 years from the date of cancer surgery.

**Table 1.** Clinical characteristics of the study population.

Characteristic	*n* = 147
Age	
Mean (SD)	56.1 (10.9)
Median (Q1, Q3)	54.7 (48.3, 62.9)
Range (min, max)	(32.3, 83.7)
BMI	
Mean (SD)	26.5 (4.7)
Median (Q1, Q3)	26.2 (22.9, 29)
Range (min, max)	(17.7, 41.9)
Missing	24
Mammographic breast density (BI-RADS)	
Almost entirely fatty (A or 1: <25%)	8 (7)
Scattered areas of fibroglandular density (B or 2: <50%)	48 (44)
Heterogeneously dense (C or 3: >50%)	45 (41)
Extremely dense (D or 4: >75%)	9 (8)
Missing	37
T category	
Tis	31 (21)
T1	73 (50)
T2	36 (24)
T3	7 (5)
*N* stage	
Nx	19 (13)
N0	91 (62)
N1	31 (21)
N2	4 (3)
N3	2 (1)
Number of pathologic nodes	
Mean (SD)	0.9 (2.5)
Median (Q1, Q3)	0 (0, 1)
Range (min, max)	(0, 19)
Number of nodes removed	
Mean (SD)	5.4 (7.1)
Median (Q1, Q3)	2 (1, 7)
Range (min, max)	(0, 35)
Primary surgery	
Lumpectomy	131 (89)
Mastectomy	16 (11)
Axillary surgery	
None	31 (21)
Sentinel lymph node biopsy (SLNB)	82 (56)
ALND	34 (23)
Chemotherapy	
None	95 (65)
Neoadjuvant	12 (8)
Adjuvant	40 (27)
Hormone therapy	
No	53 (36)
Yes	94 (64)
Radiotherapy volume and fractionation	
Local-hypofractionation	101 (69)
Local-conventional fractionation	11 (7)
Locoregional-conventional fractionation	35 (24)
Radiotherapy boost	
No boost	76 (52)
Boost	71 (48)
Lymphedema presence	
No	132 (90)
Yes	15 (10)
Follow-up time (years)	
Median (Q1, Q3)	4.3 (2.6, 7.4)
Range (min, max)	(0.2, 11.8)

### Cytokine prioritization

Using our established clinical model (21), the study population was stratified into clinically low-risk (*n* = 121) and clinically high-risk (*n* = 26) groups. Wilcoxon rank-sum testing was conducted between these two groups for each of the 17 candidate cytokines to identify those cytokines that are independent from known clinical factors (Table 2). Cytokines demonstrating significant association with the known clinical risk groups (CRP, IL1β, IL1RA, IL4, IL6, IP10, MCP1, TNFRII, and TNFα) were identified as redundant with the clinical data and therefore eliminated from further analysis. Cytokines without significant association with the known clinical risk groups (IFNγ, IFNα2A, IL10, IL17A, MMP2, MMP9, SDF1α, and TGFβ1) were classified as independent and considered for further evaluation.

**Table 2.** Identification of redundant cytokines.

​	Clinically low risk (*n* = 121)	Clinically high risk (*n* = 26)	*P* value
CRP			**<0.001**
Mean (SD)	5,544,675.9 (14,857,482.2)	11,808,917.7 (16,157,727.7)	​
Median (Q1, Q3)	2,004,950 (936,682.7, 4,048,530.2)	5,656,149.6 (2,931,687.5, 11,712,601.8)	​
Range (min, max)	(75,201.2, 108,604,645.1)	(464,254.2, 72,167,262.6)	​
IFNγ			0.63
Mean (SD)	14.5 (20.3)	14.3 (17.5)	​
Median (Q1, Q3)	9.5 (6.2, 15.3)	8.8 (5.4, 14.7)	​
Range (min, max)	(1.6, 176.7)	(3.6, 85.9)	​
IFNα2A			0.21
Mean (SD)	1.2 (0.5)	1.4 (0.5)	​
Median (Q1, Q3)	1.2 (0.9, 1.5)	1.4 (0.9, 1.9)	​
Range (min, max)	(0.3, 2.7)	(0.3, 2.2)	​
IL10			0.20
Mean (SD)	0.3 (0.4)	0.5 (0.7)	​
Median (Q1, Q3)	0.3 (0.2, 0.4)	0.3 (0.2, 0.5)	​
Range (min, max)	(0, 4.6)	(0.1, 4)	​
IL17A			0.12
Mean (SD)	5.3 (3.1)	5.9 (2.4)	​
Median (Q1, Q3)	4.6 (3.4, 6.4)	5.8 (4.7, 7.2)	​
Range (min, max)	(0.5, 20.6)	(1.7, 11)	​
Missing	1	0	​
IL1β			**0.029**
Mean (SD)	0.1 (0.3)	0.1 (0.1)	​
Median (Q1, Q3)	0.1 (0.1, 0.1)	0.1 (0.1, 0.2)	​
Range (min, max)	(0, 2.3)	(0, 0.6)	​
Missing	8	0	​
IL1RA			**0.003**
Mean (SD)	293.6 (142.4)	492.8 (350.8)	​
Median (Q1, Q3)	254.8 (210.7, 352)	384.7 (239.7, 667.1)	​
Range (min, max)	(107.3, 891.8)	(131.4, 1,689.2)	​
IL4			**0.029**
Mean (SD)	0.1 (0.1)	0.1 (0)	​
Median (Q1, Q3)	0.1 (0.1, 0.1)	0.1 (0.1, 0.1)	​
Range (min, max)	(0, 0.5)	(0, 0.2)	​
IL6			**<0.001**
Mean (SD)	1.2 (0.9)	2.2 (1.5)	​
Median (Q1, Q3)	1 (0.8, 1.3)	1.8 (1.2, 2.2)	​
Range (min, max)	(0.4, 6.2)	(0.7, 7)	​
IP10			**0.044**
Mean (SD)	631.1 (548.2)	938.3 (946.1)	​
Median (Q1, Q3)	487.9 (364.1, 682.2)	571.8 (474.8, 919.1)	​
Range (min, max)	(157.2, 4,383.4)	(274.5, 3,847.7)	​
MCP1			**0.022**
Mean (SD)	344.4 (130.4)	433.9 (190.7)	​
Median (Q1, Q3)	314 (260.5, 409.6)	421 (294.9, 556)	​
Range (min, max)	(82.2, 831)	(184.1, 929.2)	​
MMP2			0.53
Mean (SD)	126,834.3 (32,862.6)	121,152.8 (29,129.7)	​
Median (Q1, Q3)	122,858.8 (102,564, 140,855.3)	114,577.9 (107,030.2, 135,099.4)	​
Range (min, max)	(67,232.8, 213,678.4)	(67,003.3, 213,764.1)	​
MMP9			0.72
Mean (SD)	203,369.2 (133,213.7)	259,364 (417,722.9)	​
Median (Q1, Q3)	164,523.7 (112,435.8, 267,914.5)	184,501 (91,113.3, 218,304.8)	​
Range (min, max)	(38,911.7, 727,209.1)	(47,832, 2,210,355)	​
SDF1α			0.81
Mean (SD)	211.5 (270.6)	257.6 (339.5)	​
Median (Q1, Q3)	170.2 (118.3, 224.8)	172.8 (119.3, 249.9)	​
Range (min, max)	(38, 2,839.3)	(47.6, 1,829.3)	​
Missing	9	0	​
TGFβ1			0.12
Mean (SD)	17,663.6 (6,899.3)	19,458.3 (5,634.4)	​
Median (Q1, Q3)	16,919.1 (13,000.9, 21,342.8)	19,306.2 (15,326.8, 23,757.7)	​
Range (min, max)	(5,121.8, 49,386.5)	(9,819.6, 32,431.5)	​
TNFRII			**0.005**
Mean (SD)	6,171.5 (2,872.3)	8,573.5 (4,617.7)	​
Median (Q1, Q3)	5,617.9 (4,382.1, 7,472.3)	7,551.6 (5,280.7, 10,247.9)	​
Range (min, max)	(1,982.7, 22,633.7)	(3,294.6, 23,783.9)	​
TNFα			**0.019**
Mean (SD)	3.3 (1.2)	4.1 (1.6)	​
Median (Q1, Q3)	3.3 (2.6, 3.8)	4.3 (2.7, 4.5)	​
Range (min, max)	(1.1, 9.3)	(1.6, 8.5)	​

Cytokine levels are reported in pg/mL. A Wilcoxon rank-sum test was performed between candidate cytokines at baseline and established clinical risk assessment. Bolded *P* values are statistically significant (*P* < 0.05).

### Identification of top candidate cytokines for lymphedema occurrence

The eight cytokines identified as independent from known clinical risk factors based on Wilcoxon rank-sum testing were further analyzed using univariate logistic regression for lymphedema occurrence (Table 3). Odds ratios (OR) were calculated for each cytokine, and those with *P* < 0.05 (IFNα2A, IL17A, and IFNγ) were deemed to be significantly associated with lymphedema occurrence. Among these three cytokines, IFNα2A had the highest OR and was therefore selected as our top candidate for analysis of 3-year LFS and model development.

**Table 3.** Univariate logistic regression of candidate cytokines for lymphedema occurrence.

Cytokine	OR (95% CI)	*P* value	*N*
IFNα2A	3.10 (1.05–9.51)	**0.042**	147
IL17A	1.17 (1.01–1.36)	**0.027**	146
IL10	1.14 (0.29–2.41)	0.77	147
IFNγ	1.03 (1.01–1.05)	**0.021**	147
MMP2	1 (1–1)	0.86	147
MMP9	1 (1–1)	0.51	147
SDF1α	1 (0.99–1)	0.75	138
TGFβ1	1 (1–1)	0.89	147

Bolded *P* values are statistically significant (*P* < 0.05).

### IFNα2A as a predictor for lymphedema occurrence

**Figure 1.**
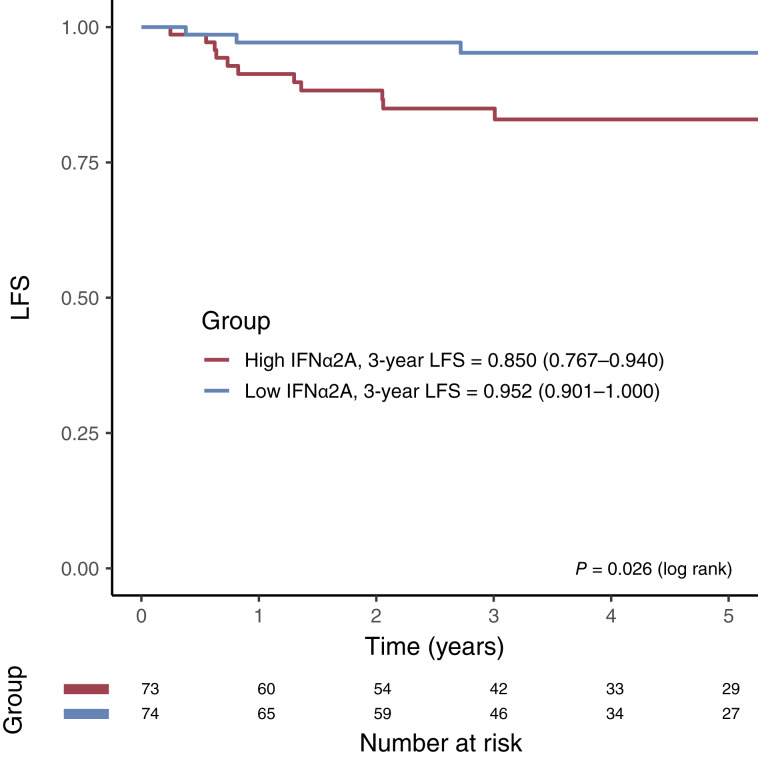
Kaplan–Meier curve of LFS by IFNα2A. A median threshold was used to define the high and low groups by IFNα2A level. Log-rank *P* value is 0.026.

We generated a Kaplan–Meier curve to assess LFS as a function of median value of IFNα2A levels (Fig. 1). At 3 years after cancer surgery, 95.2% of the low-IFNα2A group was free of lymphedema, whereas only 85% of the high-IFN-α2A group was free of lymphedema (*P* = 0.026).

**Figure 2.**
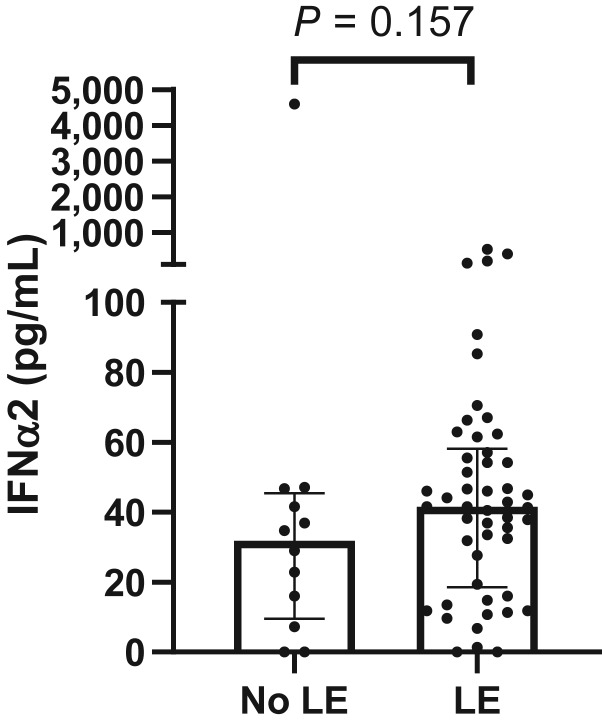
IFNα2 levels in patients with breast cancer. Preradiotherapy IFNα2 was measured in 62 patients with breast cancer. Patients were grouped by their lymphedema (LE) status at 18 months after cancer treatment. Data are plotted as median with IQR. Two-tailed *P* value is 0.157 (Mann–Whitney test).

In an effort to validate the association of IFNα2A with lymphedema, we assessed an independent cohort of 62 patients with breast cancer who were treated with lumpectomy or mastectomy in addition to ALND and radiotherapy at the University of Texas MD Anderson Cancer Center. In this cohort, 50 patients were clinically diagnosed with lymphedema, at 18 months follow-up. Similarly, plasma IFNα2 was measured prior to radiotherapy and there was a trend toward increased IFNα2 levels in patients who developed lymphedema although this trend was not shown to be significant (Fig. 2).

We then added IFNα2A to our established model, thus creating an integrated two-step clinical risk model with IFNα2A risk confirmation. The first step involves a clinical risk assessment using the five previously identified clinical risk factors of age, BMI, mammographic breast density, number of pathologic lymph nodes, and ALND, and the second step involves assessment of the IFNα2A level. We assessed the performance of this new two-step model using net reclassification improvement (Table 4). For patients assessed to be at high risk for lymphedema based on clinical factors, IFNα2A helped correctly reclassify eight of 26 patients (31%) who are actually at low risk for developing lymphedema but may continue to incorrectly classify two of 26 patients (8%) as having high risk of lymphedema. For patients assessed to be low risk for developing lymphedema based on clinical factors, IFNα2A correctly reclassified two of 121 (2%) of patients as having high risk for developing lymphedema but incorrectly reclassified 55 of 121 (45%) as being high risk.

**Table 4.** Net reclassification improvement by IFNα2a.

​	Clinical risk assessment		
	High risk	Low risk	Total
IFNα2A	​	​	​
High level	LE: 10 No LE: 6	LE: 2^a^ No LE: 55^b^	73
Low level	LE: 2^b^ No LE: 8^a^	LE: 1 No LE: 63	74
Total	26	121	147

Abbreviation: LE, lymphedema.

a Correct reclassification by IFNα2A.

b Incorrect reclassification by IFNα2A.

### Determining the additive predictive value of IFNα2A

To specifically quantify the contribution of IFNα2A to lymphedema prediction relative to the established clinical risk score, three distinct logistic regression models with combinations of these predictors were developed. All models were trained on the full cohort (*N* = 147) using continuous inputs of calculated five-factor clinical risk score and IFNα2A levels. Model A predicted lymphedema using the clinical risk score. Model B predicted lymphedema using the IFNα2A level. Model C predicted lymphedema using both clinical risk group and IFNα2A level. Both continuous predictors were standardized (*z*-score transformed) to facilitate model convergence and comparison of coefficients. A balanced class-weight parameter was used to account for the low prevalence of lymphedema (15 of 147, 10.2%), thereby permitting the calculation of informative performance metrics for the minority class. Nonparametric 95% CIs were generated using 1,000 bootstrap resamples. The performance metrics for the three models are presented in Table 5.

**Table 5.** Performance comparison of logistic regression models to determine the additive value of IFNα2A.

Model performance measures	Model A: clinical risk only	Model B: IFNα2A only	Model C: combined
Sensitivity	0.867 (0.600–1)	0.800 (0.533–1)	0.867 (0.600–1)
Specificity	0.841 (0.773–0.902)	0.553 (0.462–0.636)	0.871 (0.803–0.924)
Accuracy	0.844 (0.782–0.898)	0.578 (0.503–0.646)	0.871 (0.816–0.918)
PPV	0.382 (0.250–0.524)	0.171 (0.111–0.247)	0.448 (0.296–0.615)
Negative predictive value	0.982 (0.949–1)	0.959 (0.890–1)	0.983 (0.950–1)
AUC ROC	**0.875** (0.758–0.963)	0.672 (0.528–0.806)	**0.895** (0.796–0.971)
Brier score	**0.106** (0.066–0.155)	0.219 (0.172–0.270)	**0.101** (0.061–0.149)

Model A (clinical risk only), model B (IFNα2A only), and model C (combined). Metrics are reported as point estimate (95% CI). Bolded values compare discrimination (AUC) and calibration (Brier score) in the combined model versus clinical risk only model.

As a univariable predictor (model B), IFNα2A demonstrated poor discriminatory capacity, with an AUC of 0.672 (95% CI, 0.528–0.806). The continuous clinical risk score (model A) served as a very strong baseline, achieving an AUC of 0.875 (95% CI, 0.758–0.963).

The combined model using both IFNα2A and clinical risk score (model C) achieved the highest performance across all key metrics. Its AUC of 0.895 (95% CI, 0.796–0.971) and Brier score of 0.101 (95% CI, 0.061–0.149) both represent a favorable improvement over model A.

In threshold-dependent metrics, model C demonstrates a higher accuracy (0.871) than model A (0.844) and improved specificity (0.871 vs. 0.841) while maintaining the same high sensitivity (0.867).

Although the clinical risk score is the dominant predictor variable, this analysis indicates that IFNα2A may be useful to provide additive predictive utility, improving discrimination (AUC), calibration (Brier score), and overall accuracy in a combined model.

## Discussion

Differential cytokine expression is an important feature in the development of secondary lymphedema. In this study, we evaluated the association of 17 baseline serum cytokines with the occurrence of BCRL in a cohort of patients with mainly early-stage breast cancer, which is the most commonly presenting population of patients with breast cancer in developed countries, in order to identify key cytokine biomarkers for lymphedema prediction. We observed that IFNα2A had the greatest association with lymphedema occurrence [OR, 3.10 (95% CI, 1.05–9.51); *P* = 0.042], independent from previously established clinical risk factors. This association was evaluated in an independent cohort of patients with breast cancer from another institution, and a trend was observed toward elevated baseline IFNα2 levels in patients who went on to develop lymphedema although the trend in this second cohort was not found to be statistically significant. By incorporating IFNα2A into our established BCRL model, we were able to create an integrated two-step clinical risk model with IFNα2A risk confirmation, correctly reclassifying patients as low risk who were previously classified as high risk by clinical factors alone. Together, these results highlight the potential value of using IFNα2A to improve BCRL prognostication.

Type I interferons include IFNα, IFNω, and IFNβ, of which IFNα2A belongs to the IFNα subtype. Although the causal role of IFNα2A in BCRL has not been previously investigated, past research from existing literature has shown that IFNα inhibits lymphangiogenesis *in vitro* by suppressing lymphatic endothelial cell (LEC) growth, proliferation, and migration in a dose-dependent manner, in addition to promoting LEC apoptosis (25). The inhibition of lymphangiogenesis has been found to induce lymphedema in mice (26), suggesting a possible mechanism by which IFNα contributes to the development of BCRL.

IFNα is also known to stimulate the production of Th1 cells, which secrete the proinflammatory cytokines IFNγ, IL1β, IL2, and TNFα (27). These other cytokines induced by IFNα may also affect the progression of lymphedema through various mechanisms. The increased presence of IFNγ, IL2, and TNFα contributes to the cycle of chronic inflammation observed in lymphedema (12). In addition to its role in inflammation, IFNγ also inhibits lymphangiogenesis in a similar manner as IFN-α, by acting on LECs (25). IL1β and TNFα have been shown to dampen lymphatic pumping (2). Linking these findings in the clinical context, IFNα2A, IL1β, IL2, and TNFα have been found to be elevated in patients with lymphedema (20, 28).

In light of the relevance of IFNα in existing literature on lymphedema and its significance in our statistical analysis, we selected IFNα2A as a key cytokine biomarker to incorporate into our BCRL models. By using IFNα2A as a measure for secondary risk confirmation in our two-step model, patients who were previously identified as having high risk based on clinical factors alone were correctly reclassified as low risk by IFNα2A in 31% (eight of 26) of cases. This improvement brings forward a practical role for IFNα2A in clarifying the risk status of those labeled as high risk based solely on clinical risk factors; patients in this group could feasibly undergo baseline bloodwork to detect IFNα2A levels prior to their cancer treatment to avoid false positives based on clinical risk prediction alone.

However, it is important to note that IFNα2A may incorrectly reclassify 55 of 121 (45%) low-risk patients as being high risk. Therefore, it should not be used as a marker for risk clarification in low-risk patients. The poorer performance of IFNα2A on this low-to-high risk reclassification is likely a reflection of the outcomes of our dataset as the majority of patients in our study population did not develop lymphedema. This is one of the limitations of our study, and it is possible that the performance and thresholding for IFNα2A risk determination may be optimized using additional data.

A primary goal of this study was to determine whether pretreatment IFNα2A levels, as a continuous variable, could improve lymphedema risk prediction when added to an established clinical risk score. To isolate this effect, we compared the performance of three logistic regression models: the clinical score alone (model A), the IFNα2A level alone (model B), and a combined model (model C).

The results, presented in Table 5, provide several key insights. First, as a univariable predictor, the continuous IFNα2A level demonstrated poor discriminatory capacity [AUC 0.672 (95% CI, 0.528–0.806)], confirming that it has little-to-no diagnostic value on its own. In contrast, the continuous clinical risk score served as a strong baseline predictor, achieving a high AUC of 0.875 (95% CI, 0.758–0.963).

The central finding is that the combined model using IFNα2A in addition to the clinical risk score yielded the best performance. The point estimates for key metrics improved relative to clinical risk score alone: accuracy (0.871 vs. 0.844), specificity (0.871 vs. 0.841), positive predictive value (PPV) (0.448 vs. 0.382), AUC (0.895 vs. 0.875), and Brier score (0.101 vs. 0.106). This consistent improvement suggests that IFNα2A, even as a weak standalone predictor, provides complementary information that enhances the primary clinical model.

It is critical, however, to interpret these findings in the context of their statistical precision. The 95% CIs for the primary metrics of the univariable and combined models show considerable overlap. This overlap indicates that although the point estimates indicate performance improvement, our study is statistically underpowered to declare a definitive, significant difference between the models. This imprecision is a direct mathematical consequence of the low prevalence of lymphedema (*N* = 15) in our cohort, which results in wide CIs.

This analysis has two other important limitations. First, all models were trained and evaluated on the same 147-patient cohort. Without an independent validation set, the reported performance metrics are likely optimistic and susceptible to overfitting. The true value of this analysis is not in the absolute performance of the combined model but in the relative improvement observed over the univariable clinical risk score model.

Despite these limitations, this analysis provides quantitative evidence that IFNα2A holds utility as a complementary, rather than a primary, biomarker. It supports the two-step clinical model proposed earlier, in which the biomarker is not used for initial screening but rather to refine the risk assessment for patients already stratified by clinical factors. Future studies with larger, independent cohorts are essential to validate these findings and more precisely quantify the real-world clinical utility of IFNα2A in lymphedema risk prediction.

This study presents further unique strengths and limitations. To our knowledge, it is the first study to integrate a cytokine biomarker with clinical risk factors in prognostic modeling for lymphedema. The models we developed were informed by high-quality clinical and serum data, with lymphedema outcomes, which were uniformly assessed by trained health professionals using a standardized dictation template. Moreover, we had a large overall sample size of 147 participants comprising a broad population of patients with mainly early-stage breast cancer, which is a notable strength given that the only other comparable study in the literature had a sample size of only 40 patients (20). The 17-plex cytokine panel allowed for the assessment and comparison of multiple cytokines potentially involved in lymphedema. In particular, IFNα2 was also identified by Vang and colleagues (20) as significantly elevated at the presurgery timepoint in patients with breast cancer who went on to develop lymphedema at 12 months after radiotherapy, which supports our results. To compare to the preradiotherapy findings in our study, our secondary analysis of preradiotherapy IFNα2 levels in Vang and colleagues’s cohort also showed a trend toward elevated IFNα2 in patients who later developed lymphedema by 18 months after treatment. Although this association did not reach statistical significance (*P* = 0.157), the trend nevertheless helps to corroborate our findings. Further validation of our models will be done in the future, which will allow us to further examine the performance of these models and assess their generalizability.

It is also important to acknowledge the limitations of this study, of which outcome sample size was the primary one. Consistent with the literature, only 10.2% (15 of 147) of our patients with early-stage breast cancer went on to develop lymphedema, which may limit the generalizability and predictive ability of our models. Another weakness to consider is that our lymphedema outcomes were recorded using tape measurements of arm circumferences, whereas the study by Vang and colleagues (20) used more advanced methods by measuring lymphedema with perometry and quantifying dermal backflow with near-infrared fluorescent lymphatic imaging. However, it is noted that tape measurements may be more accessible and translatable to clinical centers globally. Finally, there are additional cytokines that may be involved in lymphedema that were not included in the 17-plex panel, which may warrant future exploration.

In summary, our study identified IFNα2A as a potentially complementary cytokine biomarker for lymphedema and demonstrated the value of using this cytokine in predictive risk models for BCRL, in conjunction with five established clinical risk factors. Notably, IFNα2A correctly reclassified 31% (eight of 26) of cases as low risk which were previously classified as high risk by clinical factors alone. Identifying low-risk patients in high–clinical risk groups is of utmost clinical importance given that recent clinical guidelines recommend escalation of preventive and prophylactic compression sleeve management and/or surgical management in high–clinical risk groups. However, these upfront strategies can be costly and burdensome if employed on all patients identified as high risk by clinical factors alone. Our literature review additionally suggests a potential role of IFNα2A in mediating lymphedema, upon which additional research would be necessary to further elucidate its mechanisms. The clinical variables used in our model are already routinely collected as part of breast cancer care, and baseline bloodwork for cytokine detection would be a feasible screening method to offer to clinically high-risk patients. Those confirmed to be at high risk can work with their healthcare providers to better tailor their cancer treatments in recognition of lymphedema as a likely side effect and to enable earlier preventive management, including compressive therapy, physiotherapy, and prophylactic surgical procedures such as lymphaticovenous anastomosis, whereas those identified as low risk by IFNα2A can be spared these burdensome and costly interventions.

## Supplementary Material

Supplementary Table 1 Table S1. Summary of baseline serum cytokine levels measured at pre-radiotherapy.

## Data Availability

The computer code is available on GitHub. Anonymized data may be shared with other researchers on request by contacting the corresponding author. Requesters will be required to sign a data-sharing agreement.

## References

[1] Vicini F , Shah C, Arthur D. The increasing role of lymphedema screening, diagnosis and management as part of evidence-based guidelines for breast cancer care. Breast J 2016;22:358–9. 26929240 10.1111/tbj.12586

[2] Aldrich MB , Sevick-Muraca EM. Cytokines are systemic effectors of lymphatic function in acute inflammation. Cytokine 2013;64:362–9. 23764549 10.1016/j.cyto.2013.05.015 PMC3771384

[3] Paskett ED , Dean JA, Oliveri JM, Harrop JP. Cancer-related lymphedema risk factors, diagnosis, treatment, and impact: a review. J Clin Oncol 2012;30:3726–33. 23008299 10.1200/JCO.2012.41.8574

[4] Cheung L , Han J, Beilhack A, Joshi S, Wilburn P, Dua A, . An experimental model for the study of lymphedema and its response to therapeutic lymphangiogenesis. BioDrugs 2006;20:363–70. 17176124 10.2165/00063030-200620060-00007

[5] Zampell JC , Aschen S, Weitman ES, Yan A, Elhadad S, De Brot Andrade M, . Regulation of adipogenesis by lymphatic fluid stasis: part I. Adipogenesis, fibrosis, and inflammation. Plast Reconstr Surg 2012;129:825–34. 22456354 10.1097/PRS.0b013e3182450b2d PMC3433726

[6] Hayes SC , Johansson K, Stout NL, Prosnitz R, Armer JM, Gabram S, . Upper-body morbidity after breast cancer: incidence and evidence for evaluation, prevention, and management within a prospective surveillance model of care. Cancer 2012;118(Suppl 8):2237–49. 22488698 10.1002/cncr.27467

[7] Montagna G , Zhang J, Sevilimedu V, Charyn J, Abbate K, Gomez EA, . Risk factors and racial and ethnic disparities in patients with breast cancer–related lymphedema. JAMA Oncol 2022;8:1195–200. 35679026 10.1001/jamaoncol.2022.1628 PMC9185510

[8] Rockson SG , Keeley V, Kilbreath S, Szuba A, Towers A. Cancer-associated secondary lymphoedema. Nat Rev Dis Primer 2019;5:22. 10.1038/s41572-019-0072-5 30923312

[9] Smoot B , Cooper BA, Conley Y, Kober K, Levine JD, Mastick J, . Differences in limb volume trajectories after breast cancer treatment. J Cancer Surviv 2016;10:772–82. 26678895 10.1007/s11764-015-0507-2 PMC4912957

[10] Brown S , Dayan JH, Kataru RP, Mehrara BJ. The vicious circle of stasis, inflammation, and fibrosis in lymphedema. Plast Reconstr Surg 2023;151:330e–41e. 10.1097/PRS.0000000000009866 PMC9881755 36696336

[11] Tabibiazar R , Cheung L, Han J, Swanson J, Beilhack A, An A, . Inflammatory manifestations of experimental lymphatic insufficiency. PLoS Med 2006;3:e254. 16834456 10.1371/journal.pmed.0030254 PMC1502157

[12] Bowman C , Rockson SG. The role of inflammation in lymphedema: a narrative review of pathogenesis and opportunities for therapeutic intervention. Int J Mol Sci 2024;25:3907. 38612716 10.3390/ijms25073907 PMC11011271

[13] Lin S , Kim J, Lee M-J, Roche L, Yang NL, Tsao PS, . Prospective transcriptomic pathway analysis of human lymphatic vascular insufficiency: identification and validation of a circulating biomarker panel. PLoS One 2012;7:e52021. 23272198 10.1371/journal.pone.0052021 PMC3525657

[14] Leung G , Baggott C, West C, Elboim C, Paul SM, Cooper BA, . Cytokine candidate genes predict the development of secondary lymphedema following breast cancer surgery. Lymphat Res Biol 2014;12:10–22. 24502445 10.1089/lrb.2013.0024 PMC3961780

[15] Avraham T , Zampell JC, Yan A, Elhadad S, Weitman ES, Rockson SG, . Th2 differentiation is necessary for soft tissue fibrosis and lymphatic dysfunction resulting from lymphedema. FASEB J 2013;27:1114–26. 23193171 10.1096/fj.12-222695 PMC3574290

[16] Di S , Ziyou Y, Liu N-F. Pathological changes of lymphedematous skin: increased mast cells, related proteases, and activated transforming growth factor-β1. Lymphat Res Biol 2016;14:162–71. 27599355 10.1089/lrb.2016.0010

[17] Baik JE , Park HJ, Kataru RP, Savetsky IL, Ly CL, Shin J, . TGF-β1 mediates pathologic changes of secondary lymphedema by promoting fibrosis and inflammation. Clin Transl Med 2022;12:e758. 35652284 10.1002/ctm2.758 PMC9160979

[18] Sano M , Hirakawa S, Suzuki M, Sakabe JI, Ogawa M, Yamamoto S, . Potential role of transforming growth factor-beta 1/Smad signaling in secondary lymphedema after cancer surgery. Cancer Sci 2020;111:2620–34. 32412154 10.1111/cas.14457 PMC7385355

[19] Vang A , Chan W, Shaitelman S, Aldrich M. Plasma cytokine levels as prognostic biomarkers and reparative microsurgery outcome measures for breast cancer-related lymphedema. J Vasc Surg Venous Lymphat Disord 2023;11:447–8.

[20] Vang AR , Shaitelman SF, Rasmussen JC, Chan W, Sevick-Muraca EM, Aldrich MB. Plasma cytokines/chemokines as predictive biomarkers for lymphedema in breast cancer patients. Cancers (Basel) 2023;15:676. 36765631 10.3390/cancers15030676 PMC9913278

[21] Kwan JYY , Famiyeh P, Su J, Xu W, Kwan BYM, Jones JM, . Development and validation of a risk model for breast cancer–related lymphedema. JAMA Netw Open 2020;3:e2024373. 33175175 10.1001/jamanetworkopen.2020.24373 PMC7658732

[22] Shi W , Misra S, Li M, Su J, Chong LP, McCuske M, . Inflammatory biomarkers, hematopoietic stem cells, and symptoms in breast cancer patients undergoing adjuvant radiation therapy. JNCI Cancer Spectr 2020;4:pkaa037. 33134822 10.1093/jncics/pkaa037 PMC7583146

[23] Lin C , Su J, Wu AJ, Lin N, Hossack MS, Shi W, . External validation of a 5-factor risk model for breast cancer–related lymphedema. JAMA Netw Open 2025;8:e2455383. 39836421 10.1001/jamanetworkopen.2024.55383 PMC11751742

[24] Altara R , Manca M, Hermans KC, Daskalopoulos EP, Brunner-La Rocca HP, Hermans RJJ, . Diurnal rhythms of serum and plasma cytokine profiles in healthy elderly individuals assessed using membrane based multiplexed immunoassay. J Transl Med 2015;13:129. 25903806 10.1186/s12967-015-0477-1 PMC4414365

[25] Shao X , Liu C. Influence of IFN- α and IFN- γ on lymphangiogenesis. J Interferon Cytokine Res 2006;26:568–74. 16881867 10.1089/jir.2006.26.568

[26] Mäkinen T , Jussila L, Veikkola T, Karpanen T, Kettunen MI, Pulkkanen KJ, . Inhibition of lymphangiogenesis with resulting lymphedema in transgenic mice expressing soluble VEGF receptor-3. Nat Med 2001;7:199–205. 11175851 10.1038/84651

[27] Lee HL , Jang JW, Lee SW, Yoo SH, Kwon JH, Nam SW, . Inflammatory cytokines and change of Th1/Th2 balance as prognostic indicators for hepatocellular carcinoma in patients treated with transarterial chemoembolization. Sci Rep 2019;9:3260. 30824840 10.1038/s41598-019-40078-8 PMC6397294

[28] de Schenquer DG , Schenquer N. Serum immunoglobulins, IL-1beta, IL-2, and IFN-gamma gamma level in patients with lymphedema treated with ortho-beta-hydroxy-ethyl rutosides (HR). Arch Med Res 2001;32:129–35. 11343810 10.1016/s0188-4409(00)00272-1

