# Supplementary material for: Identification of a Cytokine Biomarker for Prognostic Modeling of Breast Cancer–Related Lymphedema
Source: Cancer Res Commun. 2026 Mar 3;6(3):456–65. doi: 10.1158/2767-9764.CRC-25-0541 (PMC13012042; doi:10.1158/2767-9764.CRC-25-0541)
Supplement: Supplementary Table 1 — Table S1. Summary of baseline serum cytokine levels measured at pre-radiotherapy. [file crc-25-0541_supplementary_table_1_suppst1.doc]

**Appendix 1. Supplementary data**

**Table S1. Summary of baseline serum cytokine levels measured at pre-radiotherapy.** The levels of 17 serum cytokines in 147 patients were quantified in pg/mL. Mean (SD), median (Q1, Q3), and range (min, max) were calculated for each cytokine.

| Cytokine | Serum cytokine levels (pg/mL) (n = 147) |
| --- | --- |
| **CRP** |  |
| Mean (SD) | 6652637.1 (15227944.3) |
| Median (Q1,Q3) | 2608078.6 (1084061.2, 5194843.3) |
| Range (min, max) | (75201.2, 108604645.1) |
| **IFN-γ** |  |
| Mean (SD) | 14.5 (19.8) |
| Median (Q1,Q3) | 9.3 (5.8, 15.4) |
| Range (min, max) | (1.6, 176.7) |
| **IFN-α2A** |  |
| Mean (SD) | 1.3 (0.5) |
| Median (Q1,Q3) | 1.2 (0.9, 1.5) |
| Range (min, max) | (0.3, 2.7) |
| **IL-10** |  |
| Mean (SD) | 0.4 (0.5) |
| Median (Q1,Q3) | 0.3 (0.2, 0.4) |
| Range (min, max) | (0.0, 4.6) |
| **IL-17A** |  |
| Mean (SD) | 5.4 (3.0) |
| Median (Q1,Q3) | 4.9 (3.4, 6.6) |
| Range (min, max) | (0.5, 20.6) |
| Missing | 1 |
| **IL-1β** |  |
| Mean (SD) | 0.1 (0.3) |
| Median (Q1,Q3) | 0.1 (0.1, 0.1) |
| Range (min, max) | (0.0, 2.3) |
| Missing | 8 |
| **IL-1RA** |  |
| Mean (SD) | 328.8 (208.7) |
| Median (Q1,Q3) | 268.1 (211.8, 377.2) |
| Range (min, max) | (107.3, 1689.2) |
| **IL-4** |  |
| Mean (SD) | 0.1 (0.1) |
| Median (Q1,Q3) | 0.1 (0.1, 0.1) |
| Range (min, max) | (0.0, 0.5) |
| **IL-6** |  |
| Mean (SD) | 1.4 (1.1) |
| Median (Q1,Q3) | 1.1 (0.8, 1.6) |
| Range (min, max) | (0.4, 7.0) |
| **IP-10** |  |
| Mean (SD) | 685.4 (643.5) |
| Median (Q1,Q3) | 502.7 (366.9, 685.9) |
| Range (min, max) | (157.2, 4383.4) |
| **MCP-1** |  |
| Mean (SD) | 360.3 (146.2) |
| Median (Q1,Q3) | 321.2 (261.5, 427.5) |
| Range (min, max) | (82.2, 929.2) |
| **MMP-2** |  |
| Mean (SD) | 125829.4 (32212.7) |
| Median (Q1,Q3) | 119671.5 (103287.8, 140301.5) |
| Range (min, max) | (67003.3, 213764.1) |
| **MMP-9** |  |
| Mean (SD) | 213273.0 (211953.1) |
| Median (Q1,Q3) | 164532.7 (109689.7, 263979.7) |
| Range (min, max) | (38911.7, 2210355.0) |
| **SDF-1α** |  |
| Mean (SD) | 220.2 (284.0) |
| Median (Q1,Q3) | 170.2 (117.8, 228.1) |
| Range (min, max) | (38.0, 2839.3) |
| Missing | 9 |
| **TGF-β1** |  |
| Mean (SD) | 17981.0 (6710.5) |
| Median (Q1,Q3) | 17056.3 (13419.0, 22705.7) |
| Range (min, max) | (5121.8, 49386.5) |
| **TNF-RII** |  |
| Mean (SD) | 6596.3 (3358.2) |
| Median (Q1,Q3) | 5819.5 (4651.1, 7818.5) |
| Range (min, max) | (1982.7, 23783.9) |
| **TNF-α** |  |
| Mean (SD) | 3.5 (1.3) |
| Median (Q1,Q3) | 3.3 (2.7, 4.0) |
| Range (min, max) | (1.1, 9.3) |
